# Honokiol Induces Apoptosis, G1 Arrest, and Autophagy in KRAS Mutant Lung Cancer Cells

**DOI:** 10.3389/fphar.2017.00199

**Published:** 2017-04-11

**Authors:** Lian-Xiang Luo, Ying Li, Zhong-Qiu Liu, Xing-Xing Fan, Fu-Gang Duan, Run-Ze Li, Xiao-Jun Yao, Elaine Lai-Han Leung, Liang Liu

**Affiliations:** ^1^State Key Laboratory of Quality Research in Chinese Medicine/Macau Institute for Applied Research in Medicine and Health, Macau University of Science and TechnologyMacau, China; ^2^International Institute for Translational Chinese Medicine, Guangzhou University of Chinese MedicineGuangzhou, China

**Keywords:** NSCLC, KRAS, honokiol, apoptosis, cell cycle, autophagy, Sirt3

## Abstract

Aberrant signaling transduction induced by mutant KRAS proteins occurs in 20∼30% of non-small cell lung cancer (NSCLC), however, a direct and effective pharmacological inhibitor targeting KRAS has not yet reached the clinic to date. Honokiol, a small molecular polyphenol natural biophenolic compound derived from the bark of magnolia trees, exerts anticancer activity, however, its mechanism remains unknown. In this study, we sought to investigate the *in vitro* effects of honokiol on NSCLC cell lines harboring KRAS mutations. Honokiol was shown to induce G1 arrest and apoptosis to inhibit the growth of KRAS mutant lung cancer cells, which was weakened by an autophagy inhibitor 3-methyladenine (3-MA), suggesting a pro-apoptotic role of honokiol-induced autophagy that was dependent on AMPK-mTOR signaling pathway. In addition, we also discovered that Sirt3 was significantly up-regulated in honokiol treated KRAS mutant lung cancer cells, leading to destabilization of its target gene Hif-1α, which indicated that the anticancer property of honokiol maybe regulated via a novel mechanism associated with the Sirt3/Hif-1α. Taken together, these results broaden our understanding of the mechanisms on honokiol effects in lung cancer, and reinforce the possibility of its potential anticancer benefit as a popular Chinese herbal medicine (CHM).

## Introduction

Lung cancer is the most prevalent type of cancer as well as the leading cause of cancer-related mortality worldwide. It is estimated that approximately 1.8 million new lung cancer cases and1.6 million deaths occur every year (Herbst et al., 2008; Chen et al., 2016). Non-small cell lung cancer (NSCLC) accounts for 85% of all lung cancer cases (Chen Z. et al., 2014). Despite advances in early detection and standard therapies, treatment of NSCLC remains elusive (Herbst et al., 2008). The treatment and prevention of lung cancer are major un-met needs that can probably be improved by a better understanding of the molecular mechanism of the driver genes. To date, a number of driver genes have been identified, including KRAS (v-Ki-ras2 Kirsten rat sarcoma viral oncogene homolog), epidermal growth factor receptor (EGFR), anaplastic lymphoma receptor tyrosine kinase (ALK), proto-oncogene tyrosine kinase c-ROS1(ROS1) (Seo et al., 2012; Cancer Genome Atlas Research Network, 2014). Among those genes, mutation of KRAS was found in 25–30% of NSCLC (Pylayeva-Gupta et al., 2011). Recent successful targeted therapies, including the EGFR inhibitor gefitinib/erlotinib for patients with EGFR mutation (Lynch et al., 2004; Tsao et al., 2005), and ALK inhibitor crizotinib for patients with ALK rearrangements (Solomon et al., 2014).

Mutated KRAS proteins have been widely identified as potential anticancer targets, which stimulated an extensive search for small-molecule inhibitors (Cox and Der, 2010). Recently, a number of small molecules and vaccine strategies targeting KRAS have been reported (Ostrem and Shokat, 2016). Despite more than 30 years of considerable effort by researchers, effective pharmacological inhibitor of targeting KRAS signaling has not yet been identified to date, due to the lack of well-defined drug-binding pockets on the surface of KRAS protein (Ostrem and Shokat, 2016). Compared to KRAS-GTP interaction, the relatively poor binding affinity of these compounds prompted a widely held perception that oncogenic KRAS was an “undruggable” cancer target (Cox et al., 2014).

Traditional Chinese Medicine (TCM) has been widely used for several thousand years and is the most prolific source of leading compounds for drug development. It contains various natural compounds with biological activity, which are claimed to have therapeutic efficacy with minimal side effects (Liu et al., 2015). In recent years, TCM-based herbal medicines have gained increasing acceptance and aroused a great deal of interest worldwide in cancer therapy (Hsiao and Liu, 2010). Honokiol [(3’,5-di-(2-propenyl)-1,1’-biphenyl-2,2’-diol] is a natural biphenolic compound derived from an extract of seed cones and the bark of magnolia trees with anti-oxidative, anti-inflammatory and anti-tumor properties (Fried and Arbiser, 2009). Several mechanisms of honokiol have suggested that it maybe be a promising anticancer agent (Averett et al., 2014), such as the inhibition of STAT3 phosphorylation and the metastases in lung cancer cells (Pan et al., 2017), as well as the suppression of the development and progression of lung tumorigenesis by deregulating EGFR and its downstream effectors (Song et al., 2016). Honokiol also activated AMP-activated protein kinase in breast cancer cells via an LKB1-dependent pathway and inhibited breast carcinogenesis (Nagalingam et al., 2012). It reported that honokiol suppressed RAS activation by blocking RAS-dependent phospholipase D (PLD) activity, but the underlying molecular mechanisms are still unclear (Garcia et al., 2008; Fried and Arbiser, 2009). In our study, we sought to assess the *in vitro* effects of honokiol on NSCLC cell lines harboring KRAS mutations and investigate its treatment mechanism of action.

## Results

### Honokiol Inhibits Cell Proliferation and Colony Formation in KRAS Mutated Cell Lines

**FIGURE 1:**
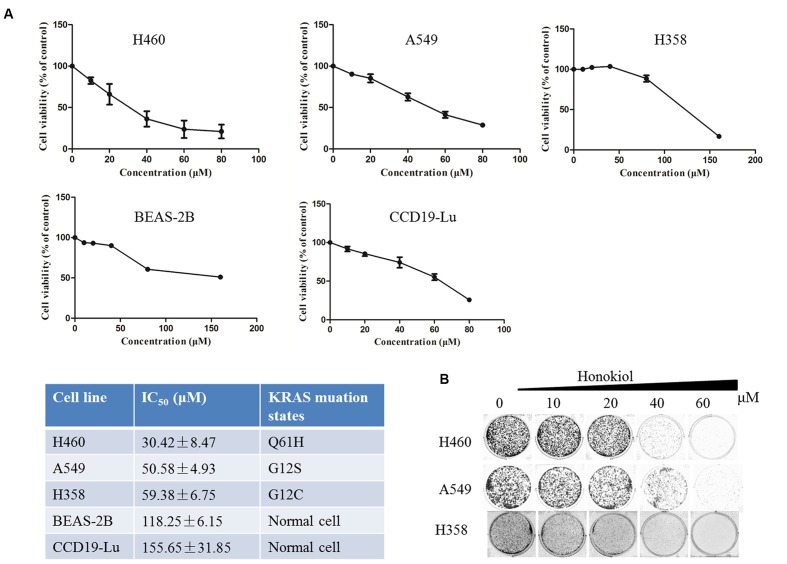
**Effect of honokiol on cell viability and colony formation in KRAS mutant cell lines. (A)** Cell viability of KRAS mutant cells and lung normal cells cultured in the presence of various concentrations of honokiol (0–80 μM) for 72 h, as analyzed by MTT assay, results were expressed as mean ± SE. **(B)** Colony formation of KRAS mutant cells was monitored after honokiol (0–60 μM) treatment for 10–14 days, and representative photomicrographs of crystal violet stained colonies were depicted.

To evaluate the therapeutic potential of honokiol, three human lung cancer cell lines H460, A549, and H358 cells were cultured with an increasing concentration of honokiol for 72 h, and then cell viability was determined by MTT assay. Honokiol inhibited the growth of H460, A549 and H358 cells in a dose-dependent manner (**Figure 1B**), with 50% inhibition concentration (IC_50_) at 72 h of 30.42 ± 8.47, 50.58 ± 4.93, and 59.38 ± 6.75 μM, respectively, but it showed low toxicity to two normal lung cells (CCD19-Lu and BEAS-2B) (**Figure 1A**). Subsequently, we examined the effect of honokiol on cell colony formation, in accordance with the cell cytotoxicity, honokiol significantly inhibited the colony formation capacity in a dose-dependent fashion in KRAS mutated cell lines (**Figure 1B**).

### Honokiol Induces Apoptosis in KRAS Mutated Cell Lines

**FIGURE 2:**
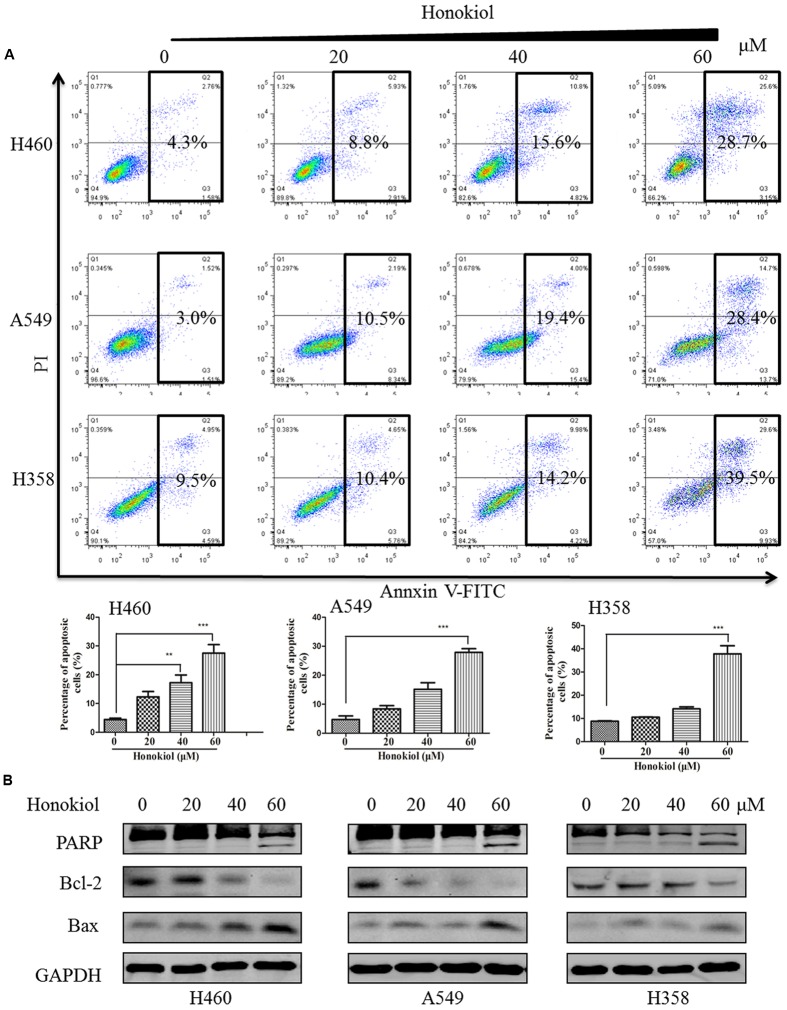
**Flow cytometry analysis of honokiol induced apoptosis in KRAS mutant cell lines. (A)** KRAS mutant cells (H460, A549, and H358) were treated with honokiol (10, 20, 40, and 60 μM) for 48 h. Cell apoptosis was analyzed using flow cytometer, triplicate data were plotted as bar chart diagram. **(B)** Cleaved PARP, Bax and Bcl_2_ protein expression was evaluated by immunoblotting of KRAS mutant cells lysates after 48 h of honokiol (10, 20, 40, and 60 μM) treatment. ^∗∗^*P* < 0.01 and ^∗∗∗^*P* < 0.001 for comparison between control group and honokiol-treated group.

To investigate whether the induction of apoptosis also contributed to honokiol-mediated growth inhibition of KRAS mutated cells, we used Annexin V-FITC/PI flow cytometry to analyze the population of apoptotic cells. Results showed that honokiol-induced apoptosis in the three KRAS mutant cell lines in a concentration dependent manner (**Figure 2A**). To further demonstrate the mechanism by which honokiol induced apoptosis in these KRAS mutant cell lines, western blotting assay was performed to evaluate the expression of several well-characterized apoptotic proteins. As shown in **Figure 2B**, honokiol increased the expression of pro-apoptotic protein Bax, while decreased the expression of anti-apoptotic protein Bcl-2 in these KRAS mutation cell lines. In addition, PARP cleavage in honokiol treated cells, further confirmed that honokiol induced apoptosis in KRAS mutated cells.

### Honokiol Suppresses Growth and Survival Signaling Pathways in KRAS Mutated Cell Lines

**FIGURE 3:**
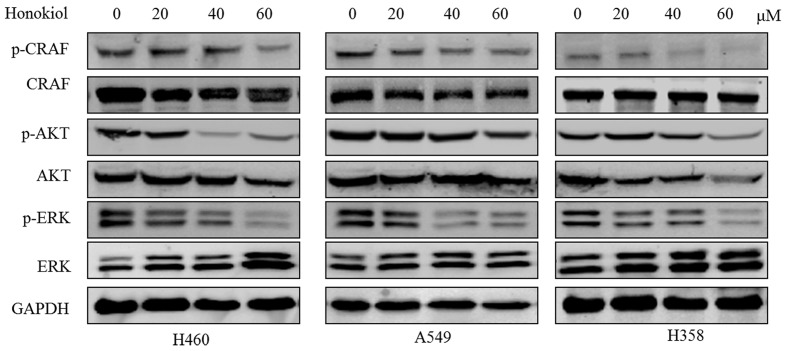
**The effect of honokiol on KRAS signaling in KRAS mutant cell lines**. KRAS mutant cells (H460, A549, and H358) were treated with 60 μM honokiol for 48 h and the levels of p-c-RAF, c-RAF, p-ERK, ERK, p-AKT, and AKT were monitored by western blotting.

Because growth factor-mediated activation of KRAS is known to activate the RAF/MEK/ERK and RAF/PI3K/AKT pathway (Castellano et al., 2013; Samatar and Poulikakos, 2014), we next examined the effect of honokiol on RAS mediated signaling transduction in KRAS mutated cells, such as the phosphorylation status of c-RAF, AKT, and ERK. The results showed that treatment with honokiol led to a reduction in c-RAF, ERK, and AKT phosphorylation in three KRAS mutated lung cancer cell lines (**Figure 3**). Our data indicated that honokiol inhibited KRAS mutated cell line growth and survival via regulating KRAS downstream signaling pathways.

### Honokiol Causes Cell Cycle Arrest at G1 Phase in KRAS Mutated Cell Lines

**FIGURE 4:**
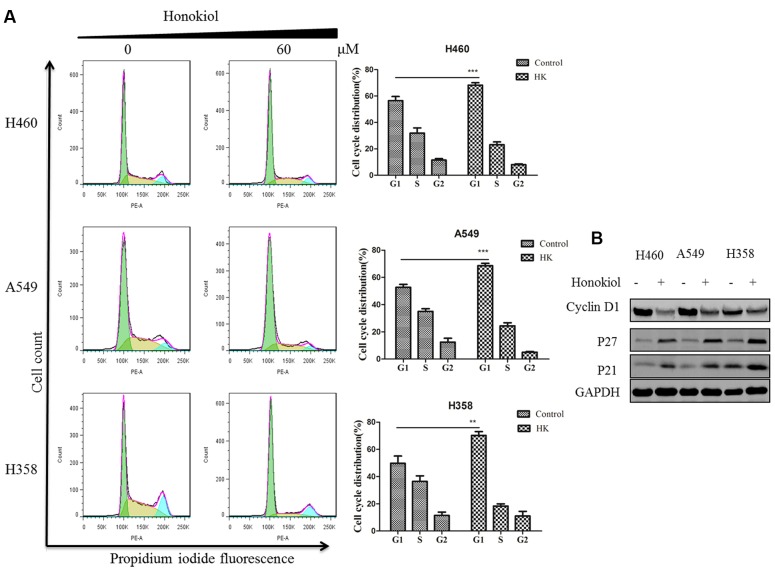
**Effect of honokiol on cell cycle progression KRAS mutant cell lines. (A)** Cell cycle distribution analysis by flow cytometry. H460, A549, and H358 cells were incubated with indicated 60 μM honokiol for 24 h. The cells were collected, stained with PI and analyzed by flow cytometer. Bar diagram showing the distribution of cells in different phases of the cell cycle. **(B)** Western blot analysis of cycle-associated proteins. KRAS mutant cells (H460, A549, and H358) were treated with 60 μM honokiol 24 h. p21, p27, and cyclin D1 protein expression was measured by western blot. ^∗∗^*P* < 0.01 and ^∗∗∗^*P* < 0.001 for comparison between control group and honokiol-treated group.

We next evaluated the cell cycle profile of KRAS mutant cells treated with honokiol. Cells (H460, A549, and H358) were treated with or without 60 μM honokiol for 24 h and cell cycle analysis was performed using flow cytometry. Results showed that treatment with honokiol led to cell cycle arrest at the G0/G1 phase in KRAS mutated cell lines (**Figure 4A**). Furthermore, we determined the effect of honokiol on cycle-related protein levels of G0/G1 phase by immunoblotting to gain insights into the mechanism of honokiol-induced cell cycle arrest in KRAS mutated cell lines. As shown in **Figure 4B**, cyclin-dependent kinase inhibitors p21 and p27 exhibited a promotion in the honokiol-treated three KRAS mutated cell lines with a marked reduction of cyclin D1. These results suggested that G1 arrest induced by honokiol might be attributed to the effect on p21, p27 as well as cyclin D1 and resulted in the inhibition of cell proliferation.

### Honokiol Induces Autophagy via mTOR-AMPK Dependent Pathway in KRAS Mutant Lung Cancer Cells

**FIGURE 5:**
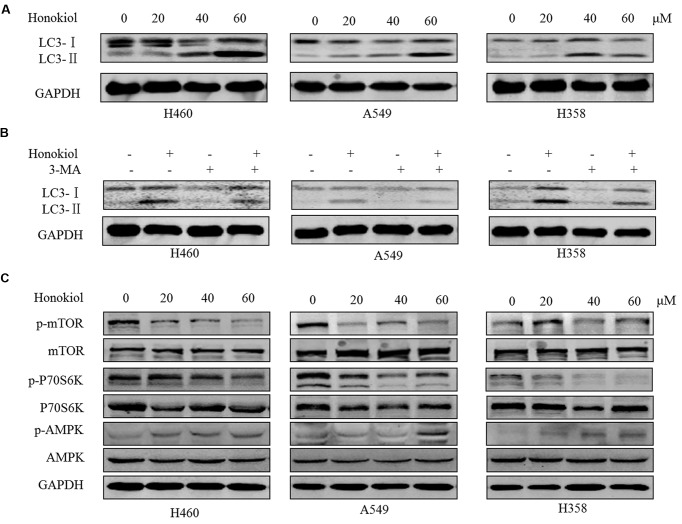
**Honokiol induced autophagy though AMPK-mTOR dependent pathway in KRAS mutant lung cancer cells. (A)** KRAS mutant cells (H460, A549, and H358) were treated with honokiol (20, 40, and 60 μM) for 24 h, the conversion of LC3-Ito LC3-II was determined by Western blot. **(B)** Cells were treated with 60 μM honokiol, 5 mM 3-MA or combination of both, the conversion of LC3-I to LC3-II was determined by Western blot. **(C)** Cells were treated with 60 μM honokiol for 24 h, p-mTOR, mTOR, p-AMPK, AMPK, p-P70S6K, and P70S6K protein expression was evaluated by immunoblotting.

Stiudies reported that honokiol can trigger autophagy (Cheng et al., 2016; Yeh et al., 2016). In order to determine whether honokiol could induce autophagy in KRAS mutated cell lines, we assessed one of the key hallmarks of autophagy: the conversion of soluble LC3-I to lipid bound LC3-II (Tanida et al., 2004). As illustrated in **Figure 5A**, treatment with honokiol distinctly increased the conversion of LC3-I to LC3-II of KRAS mutated cells in a concentration-dependent manner, and this accumulation of LC3-II induced by honokiol was relieved in the presence of autophagy inhibitor 3-methyladenine (3-MA), a class III PI3K inhibitor (**Figure 5B**). AMPK is a sensor of cellular energy status and it is activated under high intracellular AMP conditions, thereby induces autophagy via the AMPK-mTOR dependent pathway (Mihaylova and Shaw, 2011). To investigate whether the effects of honokiol on KRAS mutated cells relied on this signaling pathway, we examined the activity of honokiol on the representative signaling cascades. The results showed that treatment of cells with honokiol suppressed mTOR phosphorylation, leading to inhibition of P70S6K kinase activity, which signals cell survival and growth in transduction pathway (Asnaghi et al., 2004), with concomitant up-regulation of phospho-AMPK (**Figure 5C**). Taken together, our results suggested that honokiol induced autophagy in KRAS mutated cells was mediated through the AMPK-mTOR signaling pathway.

### Blockade of Autophagy Reduces Apoptosis in the Honokiol-treated KRAS Mutant Cells

**FIGURE 6:**
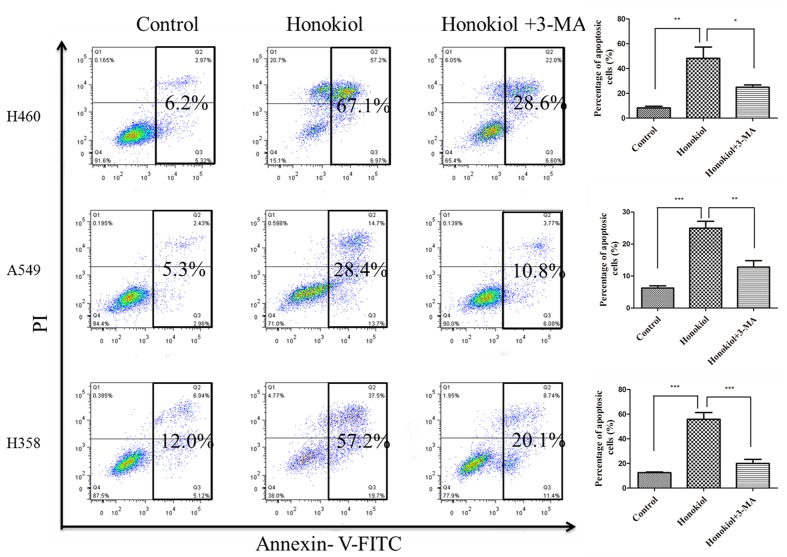
**Blockade of autophagy reduced apoptosis of the honokiol-treated KRAS mutant cells**. KRAS mutant cells (H460, A549, and H358) were treated with 60 μM honokiol, 5 mM 3-MA, or a combination of both, and cell apoptosis was measured by Annexin V/PI double staining with flow cytometry. ^∗^*P* < 0.05; ^∗∗^*P* < 0.01; ^∗∗∗^*P* < 0.001.

Although many anti-cancer agents can activate autophagy in different types of cancers, it remains controversial whether autophagy promotes cell death or acts as a pro-survival mechanism (Hippert et al., 2006). To test whether autophagy is required for honokiol-mediated growth inhibition in KRAS mutated cells, we evaluated the impact of autophagy on honokiol-mediated cytotoxicity by inhibiting autophagy with 3-MA. As shown in **Figure 6**, compared with use of honokiol alone, co-treatment with autophagy inhibitor 3-MA led to the decreased apoptosis in KRAS mutant cells, suggesting that the honokiol-induced autophagy contributed to its antitumor activity.

### Sirt3 Protein Expression is Decreased in KRAS Mutated Cell Lines While Treatment of Honokiol Increases Sirt3 Levels

**FIGURE 7:**
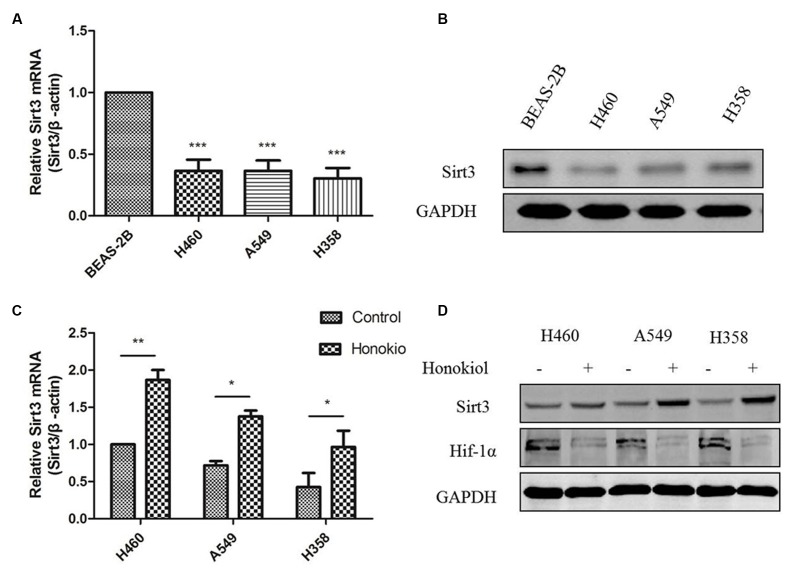
**Sirt3 protein expression was decreased in KRAS mutant cell lines and treatment honokiol increased Sirt3 levels. (A)** mRNA level of Sirt3 in KRAS mutant lung cancer cells and normal lung cell was measured by RT-PCR analysis. ^∗∗∗^*P* < 0.001, as compared to normal cells. **(B)** Cells lysate of different cell lines was subjected to immunoblotting using Sirt3 antibody. **(C)** KRAS mutant cells (H460, A549, and H358) were treated with 60 μM honokiol for 24 h and mRNA level of sirt3 was measured by RT-PCR analysis. ^∗^*P* < 0.05; ^∗∗^*P* < 0.01; ^∗∗∗^*P* < 0.001, as compared to the untreated group. **(D)** KRAS mutant cells (H460, A549, and H358) were treated with 60 μM honokiol for 24 h. Cell lysate was analyzed by western-blot with Sirt3 and Hif-1α antibodies.

Previous results have indicated that Sirt3 acts as a tumor suppressor against lung adenocarcinoma tumor tumorigenesis by maintaining mitochondrial integrity and efficient oxidative metabolism (Xiao et al., 2013; Chen Y. et al., 2014). Our data further confirmed Sirt3 mRNA levels were significantly downregulated in KRAS mutated cell lines compared with normal lung epithelial cells (**Figure 7A**). Similarly, a marked downregulation of Sirt3 protein levels was observed in KRAS mutated cell lines (**Figure 7C**). Recently, researchers discovered that honokiol might be a pharmacological activator of Sirt3 (Pillai et al., 2015), so we wondered whether honokiol can regulate Sirt3 levels in KRAS mutated cell lines. Sirt3 could mediate metabolic reprogramming in human breast cancer cells by destabilizing Hif-1α (Bell et al., 2011), The results showed that treatment of cells with honokiol increased both mRNA and protein levels of Sirt3 (**Figure 7B**), accompanied by a reduction of Hif-1a expression (**Figure 7D**), which can be destabilized by Sirt3 mediated metabolic reprogramming in human breast cancer cells (Bell et al., 2011). Collectively, these observations indicated that the anti-cancer effect of honokiol is mediated by activating Sirt3 expression.

## Discussion

**FIGURE 8:**
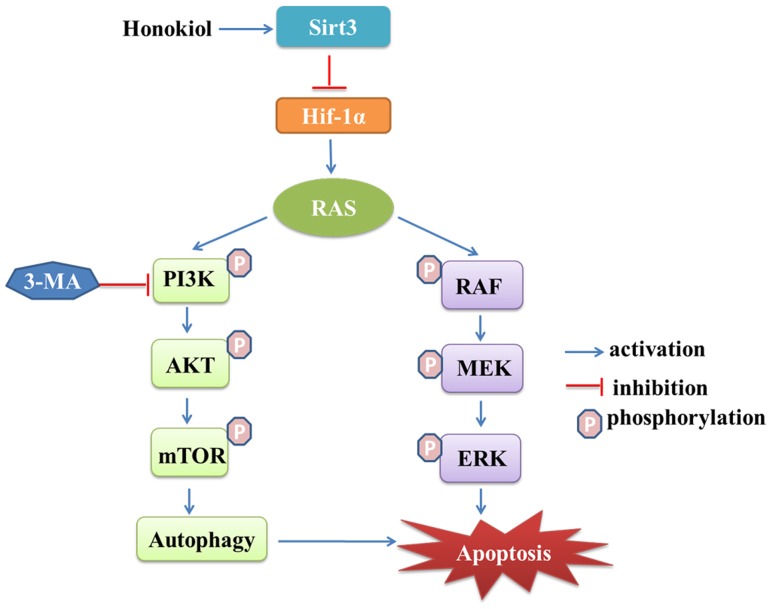
**Schematic illustration the mechanism of Sirt3 activation by honokiol**.

Honokiol has generated an extensive interest in cancer research due to its multi-functional effects such as inducing anti-tumor growth, anti-migration, anti-angiogenesis and anti-multiple drug resistance (Kumar et al., 2013; Pan et al., 2016). As a novel agent for cancer therapy, honokiol, targets multiple signaling pathways, including nuclear factor kappa B (NF-κB) (Ahn et al., 2006), signal transducers and activator of transcription 3 (STAT3), epidermal growth factor receptor (EGFR) (Leeman-Neill et al., 2010; Song et al., 2016) and mammalian target of rapamycin (m-TOR) (Lin et al., 2016), all of which have great relevance during cancer initiation and progression. In this study, we reported for the first time honokiol induced apoptosis, G1 arrest, as well as autophagy mediated cell death in KRAS mutant lung cancer cells, and honokiol blocked KRAS mutant lung cancer cells growth by activating mitochondrial Sirt3 and suppressing HIF-1α expression (**Figure 8**).

Previous studies demonstrated that, honokiol induced cytotoxicity in various human cancer cell lines (Arora et al., 2011; Avtanski et al., 2015; Zhang et al., 2015). Here, we demonstrated the cytotoxic function of honokiol in lung cancer cells harboring oncogenic KRAS for the first time. We found that honokiol induced cytotoxicity and inhibited proliferation of KRAS mutant lung cancer cells, compared to the normal lung cells with lower cytotoxicity. Cell proliferation needs cell cycle progression, which is known to be regulated positively by cyclin-CDK (cyclin dependent kinase) complexes such as cyclinB1 and cyclin D1, but negatively by CDK inhibitors such as p21 and p27 (Lacy et al., 2004; Orlando et al., 2015). Cell proliferation needs cell cycle progression. We observed that honokiol caused G1-phase arrest of cell cycle progression, which might be attributed to the reduction of cyclin D1 and enhanced expression of p21, as well as p27. Following G1-phase cell cycle arrest, cells may enter the apoptotic pathway. In the present study, we observed induction of apoptosis in honokiol-treated KRAS mutant lung cancer cells, which was supported by an increased amount of cleaved PARP and pro-apoptotic proteins Bax, with a reduction of anti-apoptotic Bcl-2 protein.

Autophagy is an evolutionarily self-digesting process in which cytoplasmic material is sequestered within cytosolic double-membraned vesicles-autophagosomes, and ends up in the lysosome (Karsli-Uzunbas et al., 2014). Autophagy has a context-dependent role in cancer, which is considered as a double-edged sword in suppression and promotion of tumorigenesis (White and DiPaola, 2009). Furthermore, recent studies suggested that autophagy is required for mitochondrial function, lipid metabolism, growth, and fate of KRASG12D-driven lung tumor (Guo and White, 2013), as well as autophagy suppresses progression of KRAS-induced lung tumors to oncocytomas and maintains lipid homeostasis (Guo et al., 2013). In our study, in KRAS mutant cells, honokiol caused the conversion of LC3-I to LC3-II, which provided an indicator of autophagic activity and could be reversed by autophagy inhibitor 3-MA, displaying its ability of inducing autophagy. Furthermore, our results revealed that honokiol-induced autophagy is dependent on the AMPK-mTOR signaling pathway, which has been addressed in autophagocytosis (Lin et al., 2016; Lu et al., 2017). Honokiol-induced autophagy may perform a pro-death process in KRAS mutant lung cancer cells, but not pro-survival response, as autophagy inhibitor 3-MA was application reduced honokiol-mediated cytotoxicity.

Sirt3, a member of the mammalian sirtin family protein that is localized to mitochondria, is a NAD^+^-dependent deacetylase and plays an important role in the control of metabolic activity (Haigis et al., 2012). Recently, studies have shown that Sirt3 acts as a tumor suppressor in lung adenocarcinoma development and progression and may be a promising therapeutic target for lung adenocarcinoma (Li et al., 2013; Akamata et al., 2016). Honokiol is a pharmacological activator of Sirt3 capable of blocking or even reversing the cardiac hypertrophic response (Pillai et al., 2015). Consistent with previous reports, we demonstrated that Sirt3 was significantly downregulated in KRAS mutant lung cancer cells compared with normal lung cells, which could be enhanced by honokiol treatment. In addition, honokiol also weakened Hif-1a expression, a Sirt3 downstream effector (Bell et al., 2011). It was reported that Sirt3 is related to autophagy, indicating the attenuation of cardiomyocytes hypertrophy by promoting autophagy (Li et al., 2016). Additionally, melatonin exerts a hepatoprotective effect on mitochondrial-derived O_2_^∙-^-stimulated autophagic cell death that is dependent on the SIRT3/SOD2 pathway (Pi et al., 2015). Therefore, our results suggested honokiol induced autophagy in KRAS mutant lung cancer cells could be attributed to Sirt3- Hif-1α pathway.

In summary, the findings here demonstrated the bioactivities of honokiol. It has a strong potential as an anticancer agent because of its ability to induce G1 arrest and apoptosis to inhibit KRAS mutant lung cancer cell growth by disrupting oncogenic KRAS-mediated RAF/MEK/ERK and RAF/PI3K/AKT signaling pathway. Importantly, honokiol triggers pro-death autophagy, which may result from the Sirt3/Hif-1a pathway. Our results thus provide a foundation for scientific and clinical development of honokiol against KRAS-driven cancers.

## Materials and Methods

### Cell Lines and Reagents

H460, A549, H358, H2122, BEAS-2B, NIH3T3, CCD19-Lu cells were obtained from the American Type Culture Collection and cultured in an environment of 5% CO_2_ at 37°C in RPMI-1640 medium supplemented with 10% fetal bovine serum (FBS), CCD19-Lu cells were grown in MEM medium supplemented with 10% FBS, BEAS-2B cells were maintained in BEBM containing 0.01 mg/ml fibronectin, 0.03 mg/ml bovine collagen type I and 0.01 mg/ml bovine serum albumin. All cells were added 100 units/ml penicillin, and 100 μg/ml streptomycin. Honokiol was purchased from Selleck Chemicals. Antibodies to GAPDH, C-RAF, p-C-RAF, p-AKT (Ser473), p-ERK (Thr202/Thy204), P21, P27, Cyclin D1, Sirt3, Hif-1α, and ERK were purchased from cell signaling technology. Anti-AKT, KRAS antibody were acquired from Santa Cruz Biotechnology.

### Cell Viability Assay

Cell viability was assayed using 3-(4.5-dimethylthiazol-2-yl)-2.5-diphenyltetrazolium bromide (MTT). Briefly, KRAS mutant cells (H460, A549, and H358) were seeded in a 96-well plate at a density of 3 × 10^3^ cells/well for 24 h, respectively, and were cultured overnight for cell adhesion, then treated with different concentrations honokiol (0–80 μM) for another 72 h. At the end of treatment, 10 μl MTT (5 mg/ml) solution was added to each well and further incubated at 37°C for 4 h, then 100 μl of the resolved solution (10% SDS and 0.1 mm HCL) was added to each well to dissolve the MTT formazan crystals. Absorbance was measured at a wavelength of 570nm with BioRad microplate reader (FluoDia T70, Photon Technology International, Lawrenceville, NJ, USA), the cell viability is calculated as the percentages change of the absorbance of treated cells divided by the absorbance of untreated cells. The IC_50_ value for the compound was determined by GraphPad Prim5.0 software.

### Colony Formation Assay

Briefly, KRAS mutant cells (H460, A549, and H358) were seeded in 6-well plates (500 cells/well), after attachment overnight, cells were exposed to various concentration of honokiol with medium changes every 3 days until visible colonies formed. The colonies were washed with cold PBS, then fixed in 4% paraformaldehyde (PFA) for 15 min, and stained with 0.5% crystal violet (1% PFA, 0.5% crystal violet, and 20% methanol in ddH2O) for 20 min. The colonies were photographed.

### Annexin V and PI Staining Assay

KRAS mutant cells (H460, A549, and H358) were treated with various concentrations of honokiol (0, 10, 20, 40, and 60 μM) for 48 h. Cells were washed with PBS and resuspended in 1× binding buffer (100 μl). One microliter of annexin V (AV)-fluorescein isothiocyanate solution (2.5 μg/ml) and 1 μl of dissolved propidium iodide (PI) (50 μg/ml) were added to the cell suspensions vortexed and incubated at room temperature in the dark for 15 min. 400 μl of chilled 1× binding buffer was added and mixed gently prior to the examination of the cell preparations by flow cytometry (FACSCalibur, BD Biosciences), a minimum of 10,000 events were collected and analyzed, and data were analyzed with the Flow J software.

### Cell Cycle Analysis

Briefly, KRAS mutant cells (H460, A549, and H358) were seeded on a 6-well plate at 1 × 10^5^ cells/well for 24 h, respectively, after treatment with 60 μM honokiol for another 24 h. The cells collected and washed with PBS, followed by fixation with 70% (v/v) ethanol at –20°C overnight. Thereafter, cells were washed and resuspended in 500 μl PBS containing 50 μg/ml PI and 1 mg/ml RNaseA for 30 min at room temperature in the dark. In total, 10,000 events were analyzed immediately in each sample subjected to flow cytometer (FACSCalibur, BD Biosciences), and the percentages of cells in G1, S and G2/M phases were calculated.

### Immunoblotting

Whole-cell lysates were rinsed with ice-cold PBS and lysed in RIPA Lysis buffer (150 mM NaCl, 50 mM Tris pH 7.4, 1 mM EDTA, 0.25% sodium deoxycholate, 1% NP-40,) containing protease (Roche) and phosphatase (Roche) inhibitors. Lysates were centrifuged at 14,100 × *g* for 10 min at 4°C. Thirty microgram of total protein from each sample was separated on a 10-12% polyacrylamide gel, and then transferred to a nitrocellulose membrane (Millipore). Immunoblots were blocked with 5% skim milk in TBS/Tween 20 (0.05%, v/v) for 1 h at RT, followed by overnight incubation at 4°C with primary anti-bodies. After washing three times with TBST, the membranes were incubated with secondary rabbit or mouse fluorescent antibodies for 1 h in the dark, then the signal intensity of the membranes was scanned on a LI-COR Odessy imaging system (Belfast). All primary antibodies were diluted in 1:1000, while their recommended secondary antibodies were diluted in 1:10000.

### RNA Isolation and Quantitative Real-time PCR

Total RNA was extracted from the cultured cells using Trizol reagent (Invitrogen) according to the manufacturer’s instructions. Total RNA (1 μg) was converted into cDNA using the PrimeScript^TM^ RT reagent kit with gDNA Eraser (Takara) according to the manufacturer’s instructions. RT-PCR was performed using SYBR^®^ Premix Ex Taq^TM^ II (Takara) in the ABI PRISM^®^ 7300 real-time PCR system (Applied Biosystems). The 2^-ΔΔCt^ method was used to calculate relative expression level.

### Statistical Analysis

GraphPad Prism 5.0 software was used to analysis data statistic. The results were presented as the mean ± standard deviation (SD). Statistical analysis was carried out using Student’s *t*-test or one-way analysis of variance. *P*-values < 0.05 were regarded statistically significant.

## Author Contributions

LL, EL, and X-JY conceived the research and led the project. EL, LL, Z-QL, and X-JY revised the manuscript. L-XL, X-XF, YL, F-GD, and R-ZL carried out the experiments and analyzed the data. EL and L-XL wrote the manuscript. All authors reviewed the manuscript.

## Conflict of Interest Statement

The authors declare that the research was conducted in the absence of any commercial or financial relationships that could be construed as a potential conflict of interest.
